# Quantitative evaluation of digital rural public sports service policies based on the PMC index model

**DOI:** 10.3389/fpubh.2025.1615982

**Published:** 2025-08-07

**Authors:** Hong Tan, Shijia Liu, Kunyu Li

**Affiliations:** ^1^College of Physical Education and Health, Southwest University of Science and Technology, Mianyang, China; ^2^School of Economics and Management, Southwest University of Science and Technology, Mianyang, China

**Keywords:** digital villages, public sports services, policy evaluation, policy quantification, PMC

## Abstract

With the continued advancement of China’s rural revitalization strategy, the high-quality development of rural public sports services has become a key priority in promoting farmers’ health and enhancing social cohesion. The rapid expansion of digital technologies offers new opportunities to overcome longstanding challenges in rural public sports provision. However, the effectiveness of current policy implementation remains uneven and faces multiple constraints. This study applies the Policy Modeling Consistency (PMC) index model to quantitatively evaluate nine representative policies, scoring and analyzing each individually. The findings reveal that only one policy is rated as excellent, five as good, and three as acceptable, with an overall average PMC score of 5.646. These results indicate that while current policies have begun to direct public sports resources toward rural areas, deficiencies remain in terms of implementation strength, coverage scope, and long-term sustainability. Based on these insights, the study recommends enhancing policy incentive mechanisms, improving the adaptability of policy tools, and establishing a cross-sectoral coordination framework to support more integrated and equitable development of urban–rural public sports services.

## 1 Introduction

Rural sports are one of the critical aspects of the national fitness campaign, yet they are also the weakest link due to the unbalanced development of sports between urban and rural areas, which is directly manifested in the scarcity of sports venues and facilities in rural areas and farmers’ generally low awareness and participation in sports activities (1). Driven by the rural revitalization strategy in the new era and the national fitness programme, achieving the equalization and high-quality development of urban and rural public sports services has already become the core issue that the current academic community and policymakers focus on (2). Public sports services are not only related to the health and well-being of rural residents and social harmony but also form an essential part of the “Healthy China” strategy and are the fundamental link in the integrated development of urban and rural areas. Nevertheless, the allocation of resources in urban and rural public sports services has long shown a significant imbalance. In particular, the backwardness of rural areas in terms of sports infrastructure construction, service levels, and resource distribution has seriously restricted the overall quality of life and physical and mental health of rural residents and hindered the in-depth progress of social fairness (3).

From the perspective of shared prosperity, sports’ role in helping rural revitalization has undergone a historical evolution from “seeking prosperity through revolution” to “getting rid of poverty and achieving common prosperity,” demonstrating multiple logical transformations in aspects such as goals, values, and models (4). To break the dilemma of the uneven allocation of urban and rural sports resources, the academic community has explored the rational allocation and equalization paths of urban and rural sports resources from multiple perspectives in recent years. Guo Xiujin et al. (5) believe that strengthening the flow and sharing of high-quality urban sports resources in rural areas is a crucial strategy to alleviate the uneven distribution of urban and rural sports resources. This promotes the complementary advantages of resources and injects new impetus into integrating urban and rural cultures (6). In addition, equalizing public sports services is the key to improving national fitness, and it is also the fundamental guarantee that urban and rural residents can share modern sports services (7). Although digital technology has mechanism support and empowerment potential in rural public sports services, it faces common problems such as lagging infrastructure, insufficient awareness of grassroots technology, poor sharing of data and information, lack of optimization of service processes, and unbalanced resource allocation. These factors have hindered the high-quality development of rural public sports services (8, 9).

The leap of new quality productive forces endows rural sports with digital capabilities (10). It breaks free from the constraints of traditional physical spaces, offers convenient and efficient sports service provisions in remote regions, and breathes unprecedented vitality into rural sports (9). The rapid progress of digital technology has furnished rural sports services with novel momentum and has emerged as a potent accelerator for high-quality development (11). For instance, digital platforms have not only remarkably enhanced the efficiency and breadth of content in rural public sports services (12) but also constructed brands with distinct local characteristics via the integrated model of “sports + culture.” This has enabled precise matching between supply and demand and optimize resource allocation, thereby infusing rural revitalization with profound cultural significance and economic dynamism (13). Nevertheless, during digital empowerment in rural sports services, practical challenges remain that demand urgent resolution, such as frail infrastructure, a scarcity of digital professionals, and an immature governance mechanism (14).

At the national level, a series of policy documents, such as the Outline of the “Healthy China 2030” Plan, have been successfully issued, clearly showing the government’s growing concern about rural public sports services. The fundamental objective of implementing these policies is to define clear goals, facilitate the balanced allocation of sports resources between urban and rural areas, and comprehensively enhance the quality of sports services in rural regions. However, in the practical implementation process, there are still numerous challenges as to whether the current policies can adequately meet the demands of rural areas and achieve a balanced development between urban and rural areas. Therefore, further in-depth research and evaluation are urgently needed. In light of this context, this study employs the PMC index model to assess the policies of digital rural public sports services quantitatively. By doing so, it aims to disclose the deficiencies of these policies and offer data support as well as scientific guidance for policy optimization, with the ultimate goal of promoting the equalization of sports services between urban and rural areas and realizing the high-quality development of rural sports services.

## 2 Original research

This study constructs a PMC index model to quantitatively assess digital rural public sports service policies, and finds that these policies have achieved initial results in directing public resources to rural areas, but there is still room for improvement in terms of implementation strength, coverage and sustainability.

## 3 Research design

### 3.1 PMC index model construction and evaluation process

The PMC (Policy Modeling Consistency) index model is a policy evaluation model that comprehensively considers various policy indicators. The establishment logic is that there is a relationship between the policy to be evaluated and any variable (15), and the role of any relevant variable should be comprehensively considered. Its significance lies in comparing the policy text with the indicators in the constructed system, intuitively presenting the elements contained in the policy, reflecting the formulation effect of the studied policy to deeply grasp the advantages and disadvantages of the formulation of the policy text on this topic.

The main steps for establishing this model are as follows:

1. Variable identification and indicator setting.
2. Establishing a multi-input–output table.
3. Calculating the PMC index.
4. Drawing the PMC surface chart.

### 3.2 Sources of texts

1. Policy Text Collection Approaches: By employing “Digital Countryside,” “National Fitness,” “Sports Public Service,” “Rural Sports,” and “Rural Revitalization” as search keywords, a search is conducted within the PKU Law and Regulations Database. Additionally, policy documents about national fitness, public service, and rural revitalization are gathered from the official websites of the General Administration of Sport of China and the National Development and Reform Commission (now abbreviated as “NDRC”), as well as from the Gazette of the State Council. Searches are also carried out in search engines like Baidu and Bing (domestic version) to ensure comprehensive coverage and fill any potential gaps.
2. Standards for Policy Text Screening: Firstly, about the issuing entity, this research exclusively opts for administrative regulations promulgated by the Party Central Committee, the State Council, the NDRC, and the General Administration of Sport of China as representative policies, while local particular digital rural public service policies are excluded from the scope of this study. Secondly, regarding the issuing content, after eliminating texts centered around the sports industry, school sports, and competitive sports, texts of the nature of annual work summaries and meeting notices are further excluded.
3. Methodologies of Policy Text Screening: The purposive sampling method and the expert interview method are utilized. The purposive sampling method is employed when the researcher has a relatively profound understanding of the research domain and can obtain policy texts with a high degree of representativeness. To enhance the representativeness and reliability of the sample selection, following purposive sampling, two rounds of opinion consultations are conducted with four experts in related fields, among whom three are from the field of national fitness and one is from the field of policy evaluation.
4. Representative Policy Determination Procedure: The sample size for policy evaluation based on the PMC model typically ranges from 8 to 12 items (16, 17). Consequently, this research has selected nine representative policies for digital rural public sports services, as presented in Table 1.

**Table 1:** Representative policies for digital rural public sports services.

Policy number	Name of policy	Release time	Thrust	issuing authority
P1	*Name of Policy: Action Programme on Strengthening Efforts to Promote the Formation of a Strong Domestic Market by Completing Shortcomings, Strengthening Weaknesses and Improving the Quality of Public Services in the Social Sector*	January 2019	Priority will be given to addressing the shortcomings of basic public services, promoting the construction and opening of public sports facilities, and accelerating the application of new-generation information technology, such as big data, cloud computing, and artificial intelligence, in radio and television transmission.	National Development and Reform Commission, Central Propaganda Department, Ministry of Education, etc.
P2	*Standards of basic public services for national fitness (2021 version)*	January 2021	It is stipulated that public sports facilities in rural areas should be open free of charge or at a low cost, that scientific fitness guidance and equipment should be provided, that there should be 1.91 instructors per 1,000 people, and that more than one mass fitness activity should be held in administrative villages each year, to ensure fair enjoyment of fitness services for all.	General Administration of Sport, in conjunction with the National Development and Reform Commission, the Ministry of Finance, the National Health Commission and the Ministry of Emergency Management
P3	*Law of the People’s Republic of China on Promotion of Rural Revitalisation*	April 2021	Improving and perfecting the network of public cultural and sports facilities and service operation mechanisms in villages and encouraging the development of diverse forms of mass cultural and sports activities for farmers.	The Standing Committee of the National People’s Congress.
P4	*Opinions on Building a Higher Level of Public Service System for Fitness for All*	March 2022	Strengthening the national fitness system, establishing a balanced fitness public service network in urban and rural areas, strengthening the equipping of townships and streets with fitness venues and equipment, enhancing community fitness activities through nighttime ‘lighting projects,’ and constructing a network of multilevel fitness facilities and a 15-min fitness circle in urban communities.	General Office of the Central Committee of the Communist Party of China and the State Council
P5	*Guidelines on Promoting the High-Quality Development of Farmers’ Sports in the 14th Five-Year Plan*	June 2022	Promote the construction and application of intelligent fitness facilities, establish a national fitness information service platform, provide fitness guidance and information services through digital means, and use a variety of platforms, such as radio and television, the Internet, and new media, to promote fitness knowledge and increase farmers’ awareness of and motivation to participate in sports.	Ministry of Agriculture and Rural Affairs, General Administration of Sport, National Rural Revitalisation Authority
P6	*Circular of the General Office of the State Council on the issuance of the Fourteenth Five-Year Plan for the Construction of Urban and Rural Community Service Systems*	November 2021	They are expanding the supply of public services such as culture and science popularisation, integrating community sports service resources to co-ordinate the construction of fitness venues and facilities for the whole population, and achieving full coverage of 15-min fitness circles in the community. Modern information technologies such as fifth-generation mobile communications (5G) and the Internet of Things are used to promote the construction of information infrastructure for intelligent communities.	State Council Office of the People’s Republic of China
P7	*Guiding Opinions on Promoting Sports for Rural Revitalisation Work*	May 2023	Serving the development of rural sports by promoting information technology and digital communication. Optimizing the construction of sports facilities, expanding fitness guidance platforms, and increasing the number of sports events and cultural activities will facilitate the allocation of rural sports resources, increase participation and sports awareness, and promote the high-quality development of rural sports.	State General Administration of Sport, Development and Reform Commission, Central Committee of the Communist Youth League, Ministry of Education and other 12 departments
P8	*Guidance on Accelerating the Digital Enablement of Life Services*	December 2023	Enhance the digitalization of public sports services and build an intelligent operation system for public stadiums.	Ministry of Commerce, National Development and Reform Commission and other 12 departments
P9	*Guide to Building Digital Villages 2.0*	April 2024	Promoting information technology to assist the development of rural sports and launching several rural sports tourism boutique routes and hotspots. Encouraging reliance on mainstream media. Making full use of network live broadcasts, short videos, and other platforms to create “rural sports people,” “rural sports net red,” and different columns, spreading positive energy in rural sports.	Central Internet Information Office, Ministry of Agriculture and Rural Development

### 3.3 Research plan design

Step 1: Familiarize with the content and characteristic structure of policies. Thoroughly study the collected policy texts of digital rural public services.
Step 2: Construct the PMC index model for policy quantification. First, evaluation criteria should be established based on Ruiz Estrada’s (15) policy evaluation research. Based on the Omnia Mobilis hypothesis, assume that all variable weights are the same when conducting policy analysis and fully incorporate relevant indicator variables.
Step 3: Policy content analysis. Adopt a combination of text mining and manual assistance to extract core keywords and conduct content analysis as the entry point of policy research.
Step 4: Conduct multi-input–output analysis. Multi-input–output analysis is a framework that can store multiple data types and measure variables with multi-dimensional indicators.
Step 5: Present a visual graph group. Intuitively present each variable as a PMC three-dimensional surface graph, compare the horizontal and vertical dimensions of policies and indicator variables, analyze the shortcomings of digital rural public service policies, and propose solutions and development directions.

## 4 Analysis and quantitative evaluation of digital rural public service policy content

### 4.1 Keyword frequency statistics

First, the national fitness public service policy texts should be imported into the text mining database of ROST CM6.0 software. Compare with the “Subject Thesaurus of the State Council Documents” and the software’s thesaurus, and preprocess the collected policy texts. To achieve the expected results, add a custom dictionary to the original dictionary to improve word segmentation accuracy. Extract the keywords related to digital rural public services, output them in the order of word frequency, and at the same time eliminate function words and common words that are not relevant to this research, such as “work,” “in short,” etc. The statistical results are shown in Table 2.

**Table 2:** High-frequency words in policy texts.

Rankings	High frequency word	Rankings	High frequency word	Rankings	High frequency word
1	Service	21	Commence	41	Well-being
2	Villagers	22	Resource	42	People’s government
3	Construct	23	Elevation	43	Robust
4	Physical education	24	Refinement	44	Comprehensively
5	Developmental	25	Build up	45	Realise
6	Countryside	26	Organise	46	Wisdom
7	Communal	27	Public service	47	Conduct
8	Sports facility	28	City and countryside	48	Training centre
9	Push forward	29	Flat-roofed building	49	Scale up
10	Agriculture	30	Managerial	50	Qualitative
11	Carry forward	31	Incentivise	51	Rural revitalisation
12	Ramp up	32	Skill	52	Manpower
13	Peasants	33	Machine	53	Abilities
14	Exercise	34	Boost	54	Informatisation
15	Digital	35	National fitness	55	The masses
16	Digitisation	36	Data	56	Education
17	Cultures	37	Infrastructural	57	Community service
18	Societies	38	Utilise	58	Fit
19	Systems	39	Guarantee	59	Coordinate
20	Nations	40	Appliance	60	Integration

### 4.2 Determination and parameter selection

Secondly, concerning the way of setting variables by scholars such as Hu Ruochen et al. (18), Shi Lizhen et al. (19), Shi Chongyan et al. (20), Li Shenghui et al. (21), Cheng Meichao (22), and Yimsuk et al. (23) ten first-level variables and 44 s-level variables are initially formulated. The first-level variables are policy nature X1, policy failure X2, issuing authority X3, policy audience X4, policy focus X5, policy evaluation X6, incentives X7, policy function X8, policy tools X9, and policy disclosure X10. After clarifying the connotation of the first-level variables, the selection of second-level variables considers various influencing factors, and the second-level variables are of equal importance and have the same weight. The value of the second-level variables follows the distribution of [0, 1] and takes the value of 0 or 1. When the content of the policy includes or involves related variables, it takes the value of 1; if it has nothing to do with the variables, it takes the value of 0. Variable X10 is only an indicator of whether the policy is open; thus, no second-level variables are set. The evaluation indicator system and evaluation criteria are shown in Table 3. Finally, after experts’ discussion and modification, nine primary and 44 secondary variables were identified (Table 4).

**Table 3:** PMC grade evaluation table.

PMC index	0–2.99	3.0–4.99	5.0–6.99	7.0–8.99	9.0–10.0
Rating criteria	Poor	Acceptable	Good	Excellent	Perfect

**Table 4:** Variable settings of the quantitative evaluation index model for digital rural public sports service policies.

Level 1 variable	Serial number	Binary variable	Serial number	Evaluation criteria	Source or basis
Nature of the policy	Y1	Descriptions	Y1:1	Whether the content of the description is addressed, yes 1, no 0	Ruiz Estrada, 2011 (15)
		Lead (around)	Y1:2	Includes guidance, yes 1, no 0	
		Suggestion	Y1:3	Whether the content is recommendatory, yes 1, no 0	
		Supervisory	Y1:4	Regulatory function, yes 1, no 0	
		Prediction	Y1:5	Includes predictive functions, yes 1, no 0	
Policy timeliness	Y2	Long term	Y2:1	1 if in force ≥ 10 years, 0 otherwise	Modified and Delphi method based on article by Y. A. Zhang et al.
		Mid-term	Y2:2	1 if in force for 5 to 10 years, 0 otherwise	
		Short-term	Y2:3	1 if in force from 1 to 5 years, 0 otherwise	
		Temporary	Y2:4	1 if in force ≤ 1 year, 0 otherwise	
issuing authority	Y3	Department of State and General Office	Y3:1	If the policy is issued by the General Office of the State Council, it will be counted as 1, otherwise it will be counted as 0.	Modification and Delphi method based on the article of Cheng Meichao et al. (22)
		State Ministries and Departments	Y3:2	Count 1 if the policy is issued by a national ministry, 0 otherwise.	
		(sth. or sb) else	Y3:3	Count 1 if the policy is issued by another national ministry, 0 otherwise.	
Policy audience	Y4	All Rural	Y4:1	1 if policy covers all villages, 0 otherwise	Zou Kai (24)
		Local government	Y4:2	1 if policy includes digitisation of resources, 0 otherwise	
		Social enterprise	Y4:3	1 if the policy relates to digital service platforms, 0 otherwise	
		Special group	Y4:4	1 if the policy relates to digital property rights, 0 otherwise	
Policy focus	Y5	Facility Description	Y5:1	1 if the policy includes a site facilities element, 0 otherwise	
		Sports culture atmosphere	Y5:2	1 if the policy content involves the enhancement of sports culture, 0 otherwise	Based on Shizhen Huang et al. (19)
		Fitness Instructor	Y5:3	1 if the policy covers fitness instruction, 0 otherwise	
		Tournament Activities	Y5:4	1 if the content of the policy relates to tournament activities, 0 otherwise	
		Talent Cultivation	Y5:5	1 if the policy content involves talent cultivation, 0 otherwise	
		Sports Organisation	Y5:6	1 if the policy involves sports organizations, 0 otherwise	
Policy evaluation	Y6	Well-founded	Y6:1	Whether institutional standards are addressed, yes 1, no 0	Wang Jinfu et al. (25)
		Clear-cut	Y6:2	Whether publicity and promotion are involved, yes 1, no 0	
		Programme science	Y6:3	1 if policy programme smart micro perspective, 0 otherwise	
		Well planned	Y6:4	1 if the policy is well planned, 0 otherwise	
		Full and accurate	Y6:5	1 if the perspective is developed from an integrated macro perspective, 0 if it is not0	
incentives	Y7	Assessment system	Y7:1	1 if there is an evaluation system for the policy content, 0 otherwise	Chi Yong et al. (26)
		Talent incentives	Y7:2	1 if the policy includes talent incentives, 0 otherwise.	
		Tax incentives	Y7:3	1 if the policy includes tax incentives, 0 otherwise.	
		Government subsidy	Y7:4	1 if the policy includes government subsidies, 0 otherwise.	
		Special fund	Y7:5	1 if policy includes specialised funds, 0 otherwise	
		Resource sharing	Y7:6	1 if policy includes resource sharing, 0 otherwise	
Policy function	Y8	Facilitating	Y8:1	1 if the policy includes a promotional function, 0 otherwise	Modified and Delphi method based on article by Y. A. Zhang et al.
		Ramp up	Y8:2	1 if policy includes enhancements, 0 otherwise	
		Developmental	Y8:3	1 if policy includes enhancements, 0 otherwise	
		Realise	Y8:4	1 if the policy includes an implementation function, 0 otherwise	
		Scale up	Y8:5	1 if policy includes enhancement, 0 otherwise	
		Otherwise	Y8:6	1 if the policy includes other functions, 0 otherwise	
Policy tools	Y9	Compulsory	Y9:1	1 if the nature of the policy is mandatory, 0 otherwise	Modified and Delphi method based on article by Y. A. Zhang et al.
		Service-oriented	Y9:2	1 if policy is service-oriented, 0 otherwise	
		Incentive-based	Y9:3	1 if policy is incentive-based, 0 otherwise	
		Market-oriented	Y9:4	1 if policy is market-based, 0 otherwise	
Policy openness	Y10	Policy disclosure	Y3:1	1 if the policy is public, 0 otherwise	Modification and Delphi method based on articles by Y. A. Zhang, RuizEstrada and others

### 4.3 Calculation of the PMC index

According to the steps related to constructing the PMC index model by Mario Arturo Ruiz Estrada, the first step is calculating the PMC index. Firstly, the primary and secondary variables are put into the multi-input–output table; next, the secondary variables are assigned values, referring to Estrada’s assignment method, as shown in Equations 1, 2; then the specific values of the primary variables are calculated, and the calculation process is shown in Equation 3; finally, the PMC index is calculated, and the calculation method is shown in Equation 4.


(1) P:N[0∼1]

(2) P={PR:[0∼1]}

(3) $$\mathrm{Pi}[\sum_{j=1}^{n}\frac{P_{\mathrm{ij}}}{T(P_{\mathrm{ij}})}]$$

Among them i=1,2,3,⋯,n,i is a first-level variable and j is a second-level variable.


(4) $$\mathrm{PMC}=\begin{array}{c} P1[\sum_{a=1}^{5}\frac{P_{1i}}{5}]+P2[\sum_{b=1}^{4}\frac{P_{2i}}{4}]+P3[\sum_{c=1}^{3}\frac{P_{3i}}{3}]+\\ P4[\sum_{d=1}^{4}\frac{P_{4i}}{4}]+P5[\sum_{e=1}^{6}\frac{P_{5i}}{6}]+P6[\sum_{f=1}^{5}\frac{P_{6i}}{5}]+\\ P7[\sum_{g=1}^{6}\frac{P_{7i}}{6}]+P8[\sum_{h=1}^{6}\frac{P_{8i}}{6}]+P9[\sum_{k=1}^{4}\frac{P_{9i}}{4}] \end{array}$$

The policies are rated based on the score results calculated according to the PMC index formula. Different scores correspond to various levels. The rating criteria are shown in Table 3 and are divided into five levels: 9–10 points (perfect), 7–8.99 points (excellent), 5–6.99 points (good), 3–4.99 (acceptable), and 0–2.99 (poor).

### 4.4 Method of drawing PMC surface chart

The PMC surface visualizes the result matrix of each policy analysis with the aid of a surface chart. Given the symmetry of the surface, X10 is excluded during the drawing process. The drawing method involves selecting the results of the first nine principal variables, constructing a 3*3 matrix with the same number of rows and columns according to Equation 5, and then drawing a three-dimensional surface. The deeper the surface’s concave surface is, the lower the score of the relevant policy variables is; conversely, the higher the score is. In this way, the advantages and disadvantages of each policy can be observed more intuitively.


(5) $$\mathrm{PMC}=[\begin{array}{ccc} X1 & X2 & X3\\ X4 & X5 & X6\\ X7 & X8 & X9 \end{array}]$$

## 5 Empirical research

### 5.1 Sample selection

This paper selects nine digital rural policies, including Guiding Opinions on Promoting High-Quality Development of Farmers’ Sports during the 14th Five-Year Plan Period, to conduct empirical research by considering factors such as policy sources, policy natures, and policy audiences.

To ensure the representativeness and validity of the selected policy samples, this study employed a combination of expert consultation and the Delphi method. Initially, the research team compiled 21 relevant national and provincial policy documents issued between 2018 and 2023 by searching keywords such as “digital rural,” “rural revitalization,” and “public sports services” from official government portals and authoritative databases.

We then invited five experts in the fields of sports sociology, rural governance, and public policy evaluation to participate in a two-round Delphi evaluation process. In the first round, experts independently assessed each policy based on its relevance, typicality, and thematic coverage. Feedback and divergent views were summarized and shared anonymously in the second round, where experts revised their opinions to reach consensus.

Policies were selected based on the following criteria: (1) clear focus on rural public sports services; (2) inclusion of digital platforms, smart governance tools, or technical systems; (3) timeliness, representativeness, and accessibility; and (4) strategic or guiding significance within the policy system.

The numbers, names, release times, and releasing institutions of the policy samples are detailed in Table 1.

### 5.2 Policy PMC model analysis

Based on the text content analysis and the calculation method of the PMC index, the nine digital rural policies are filled into the input–output table, and the PMC indexes of each policy are calculated, respectively. The results are shown in Table 5. At the same time, this study incorporates the concept of the “Depression Index” derived from the PMC surface diagram to help identify potential weak points in policy structure. The Depression Index is not an independent numerical indicator; rather, it is derived by visually detecting prominent low-value regions (“depressions”) on the PMC surface, which result from certain variables being scored as 0. This method complements the aggregate PMC score by revealing dimension-level deficiencies that might otherwise be obscured. It enhances the model’s ability to visually identify inconsistencies or gaps in policy content.

**Table 5:** Summary of the calculation of PMC indexes for each policy of digital rural public sports services.

Serial number/Policy number	P1	P2	P3	P4	P5	P6	P7	P8	P9	Mean
Y1	0.8	0.6	0.6	1	1	0.8	1	0.6	0.8	0.8
Y2	0.33	0.33	0.33	0.33	0.33	0.33	0.33	0.33	0.33	0.33
Y3	0.33	0.67	0.33	0.33	0.67	0.33	0.67	0.67	0.67	0.51
Y4	0.25	0.75	0.5	0.5	0.75	0.5	0.75	0.75	1	0.63
Y5	0.83	0.83	0.5	1	1	0.5	1	0.17	0.5	0.70
Y6	0.8	0.8	0.4	1	1	0.4	1	0.2	0.8	0.71
Y7	0.67	0.67	0.5	0.83	0.5	0.5	1	0.33	0.5	0.61
Y8	1	1	1	1	1	1	1	0.33	0.67	0.88
Y9	0.75	0.75	0.5	0.75	0.5	0.5	0.5	0.25	0.75	0.58
PMC Score	5.77	6.4	4.67	6.75	6.75	4.87	7.25	3.63	6.02	0.80
Evaluations	Favourable	Favourable	Acceptable	Favourable	Favourable	Acceptable	Excellent	Acceptable	Favourable	–
Rankings	6	4	8	2	2	7	1	9	5	–
Depression index	3.23	2.6	4.33	2.25	2.25	4.13	1.75	5.36	2.98	–

Judging from the PMC index results in Table 5, the overall ecological performance of various policies on digital rural public sports services in China from 2019 to 2021 was relatively reasonable, and the design was relatively sound. The overall ranking was P7 > P5 > P4 > P2 > P9 > P1 > P6 > P3 > P8. From the perspective of classification by grade levels, there was 1 item at the “excellent” level, 6 items at the “good” level, and 2 items at the “acceptable” level. Local governments can reasonably formulate their respective policy implementation plans, improve infrastructure, and promote the healthy development of digital rural public sports services per the national strategic guideline of “coordinating as a whole in planning.”

### 5.3 PMC surface drawing

According to Equation 1, establish the PMC matrix for the selected digital rural public sports services. Based on the matrix results, draw the PMC surfaces, as shown in Figures 1–9.

**Figure 1:**
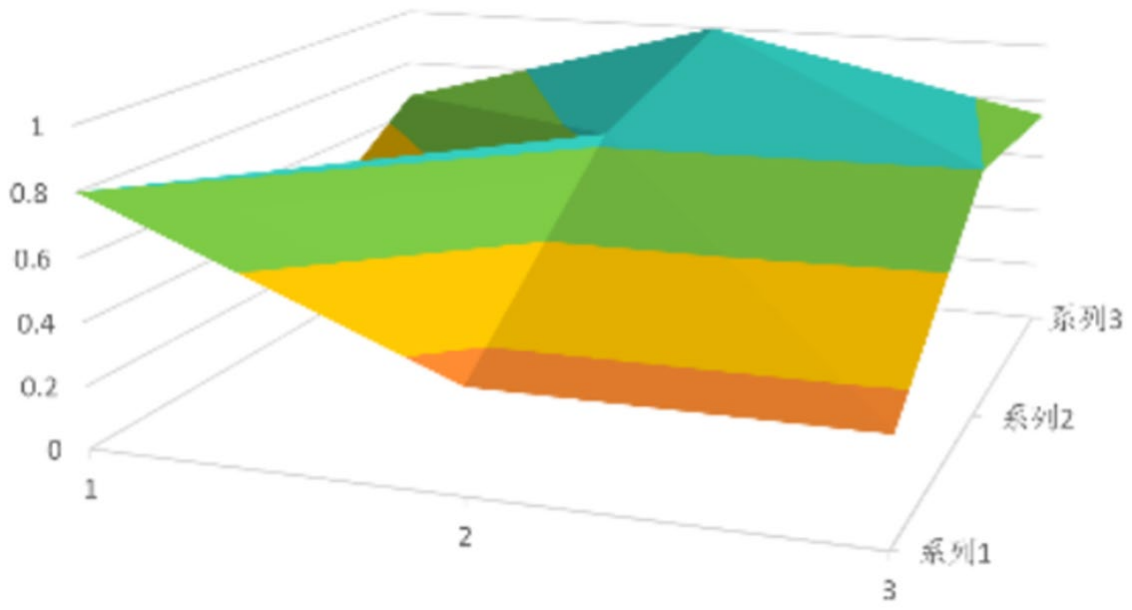
PMC profile for policy 1.

**Figure 2:**
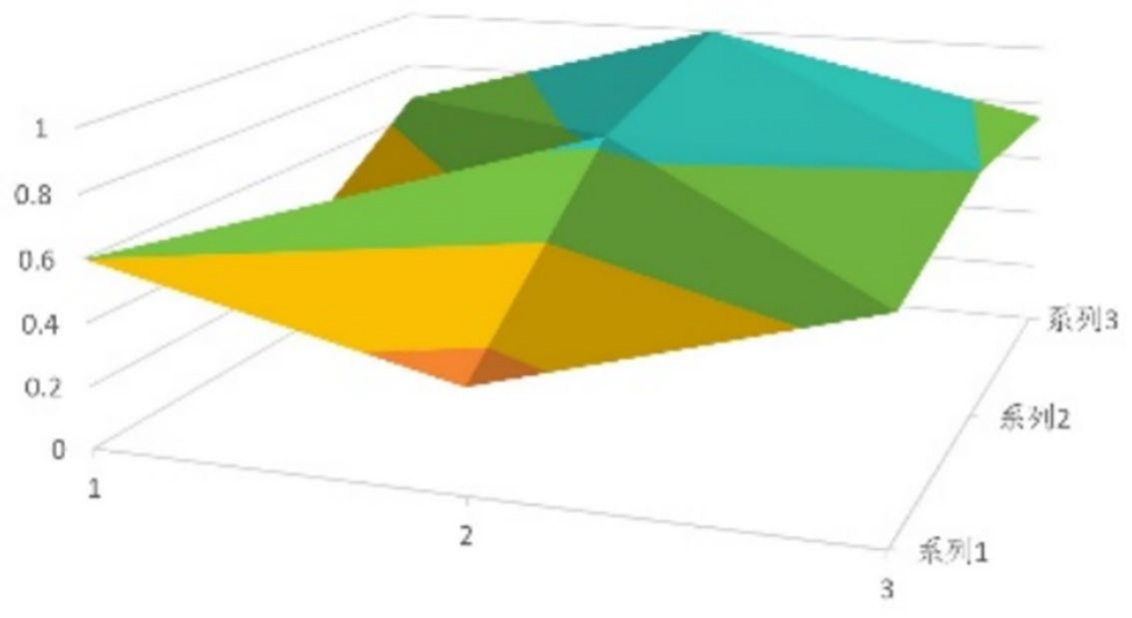
PMC profile for policy 2.

**Figure 3:**
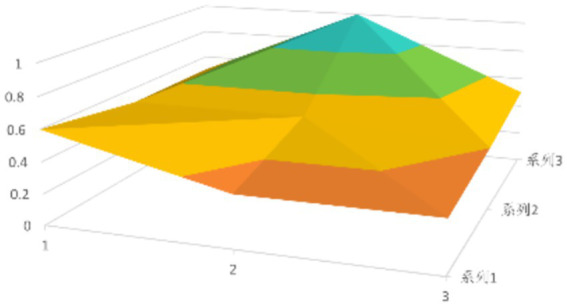
PMC profile for policy 3.

**Figure 4:**
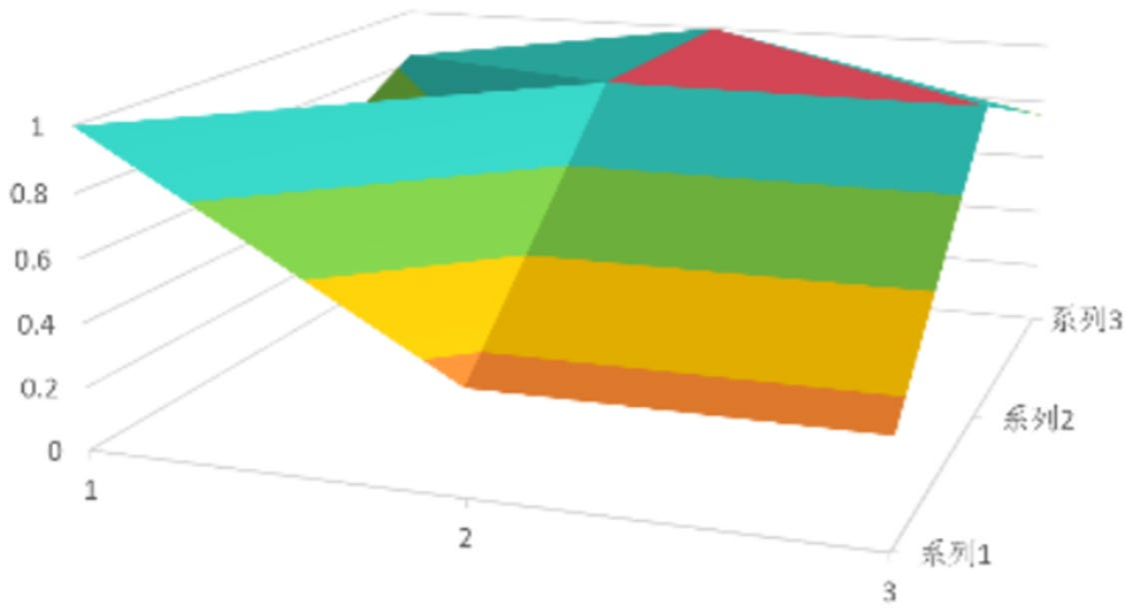
PMC profile for policy 4.

**Figure 5:**
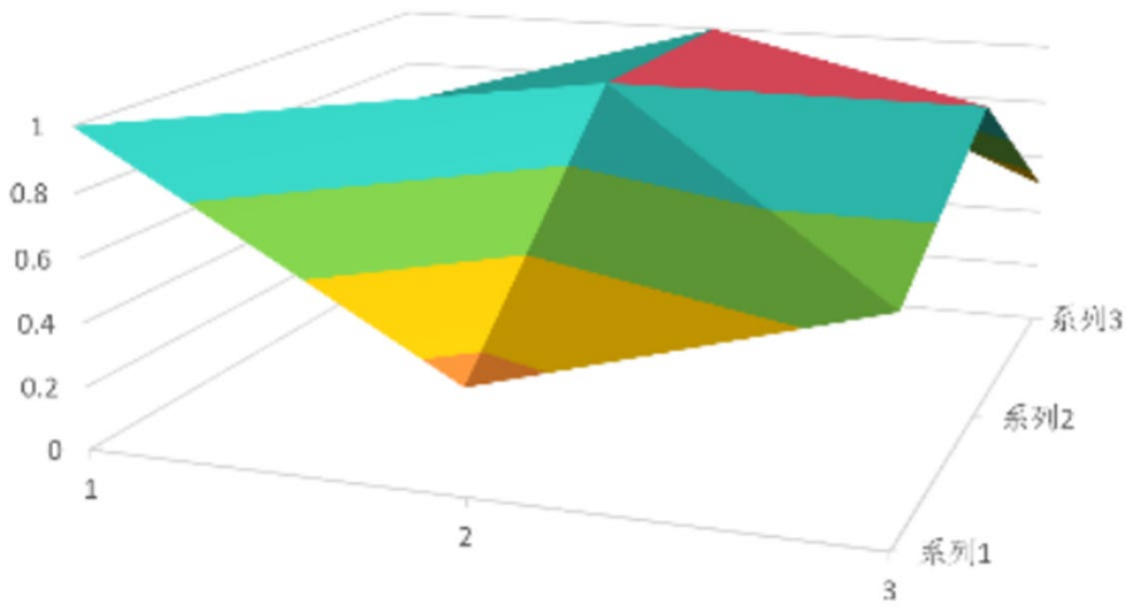
PMC profile for policy 5.

**Figure 6:**
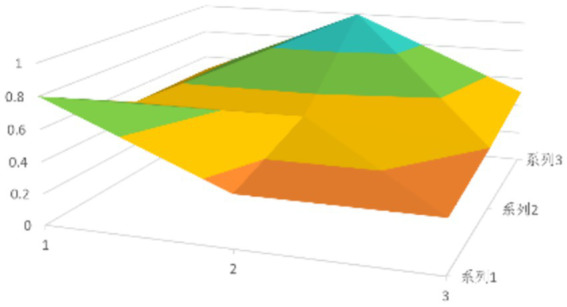
PMC profile for policy 6.

**Figure 7:**
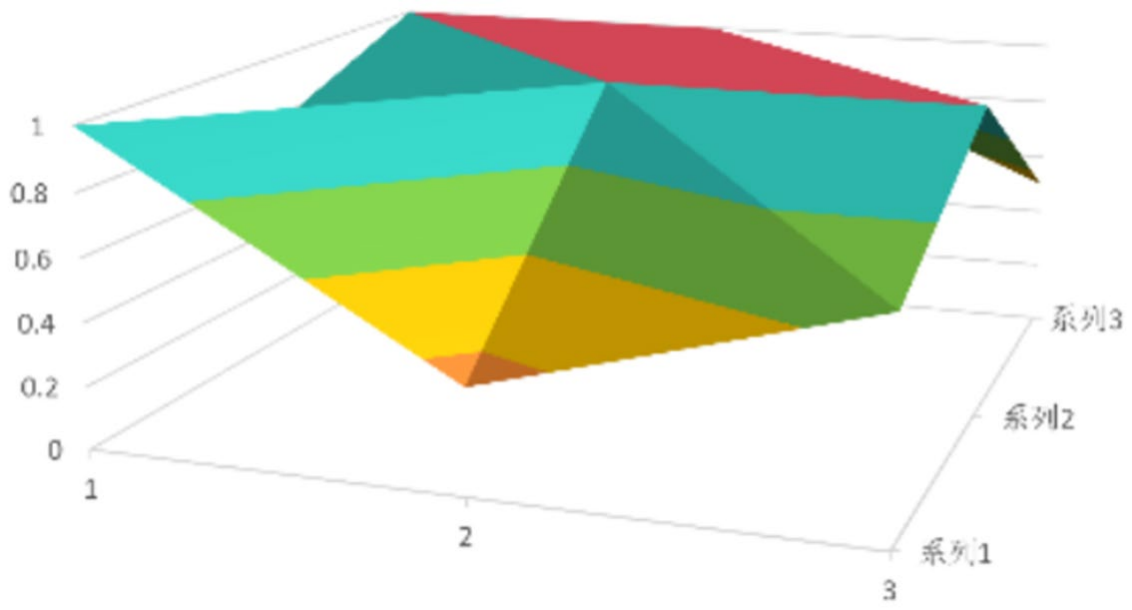
PMC profile for policy 7.

**Figure 8:**
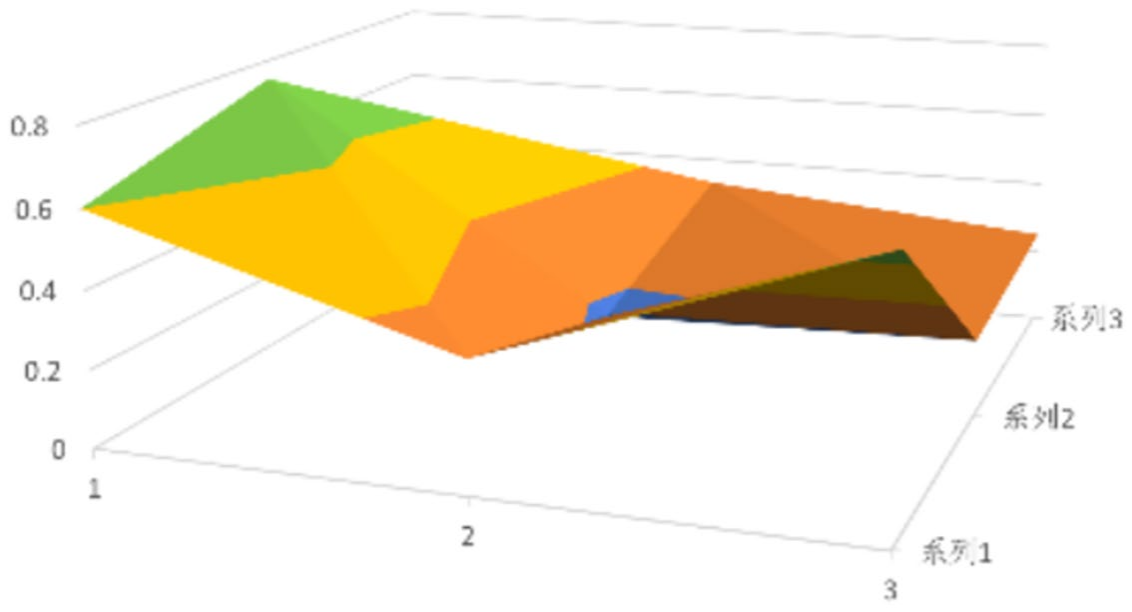
PMC profile for policy 8.

**Figure 9:**
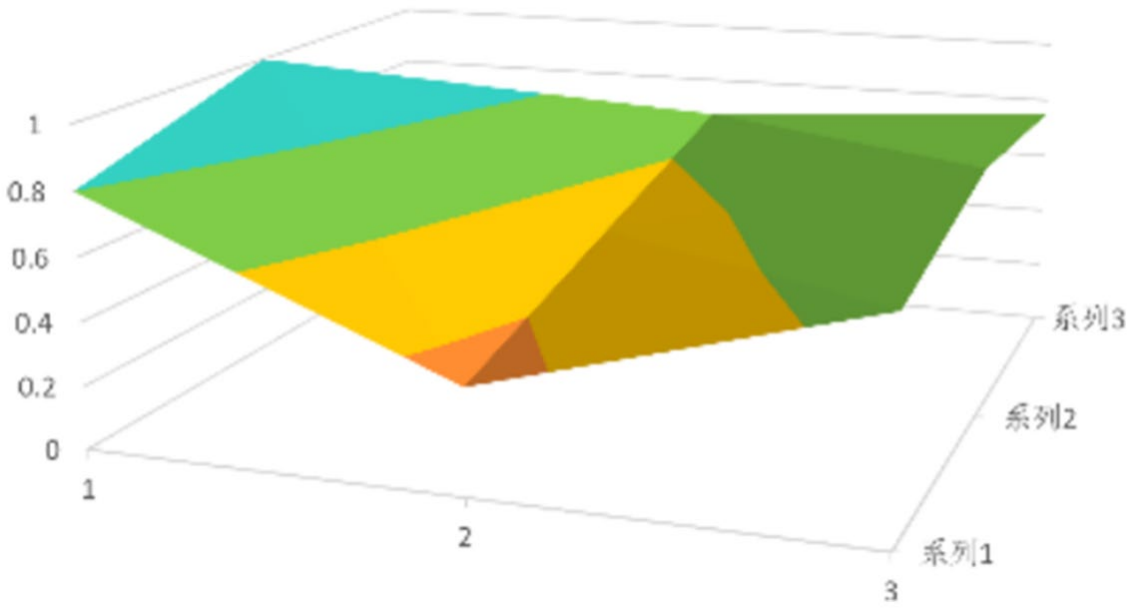
PMC profile for policy 9.

The advantages and disadvantages of each policy can be displayed intuitively by analyzing the characteristics of the PMC surfaces in the Figures 1–9. The deeper the concavity of the PMC surface is, the lower the scores of the relevant variables indicate certain deficiencies in the policy. Conversely, the closer the PMC surface is to the top of the Figures 1–9, the higher the scores of the relevant policy variables are, demonstrating more robust advantages. In addition, the PMC surface’s smoothness reflects the policy’s synergy. The smoother the surface is, the better the policy can balance different objectives. Through comparison, the PMC surfaces of policies P2, P4, P5, and P7 have relatively good smoothness (as shown in Figures 2, 4, 5, 7), reflecting that these policies possess relatively strong synergy. However, the PMC surfaces of policies P1 and P9 have relatively poor smoothness (as shown in Figures 1, 9), suggesting that these policies may have certain deficiencies when considering different objectives.

**Figure 10:**
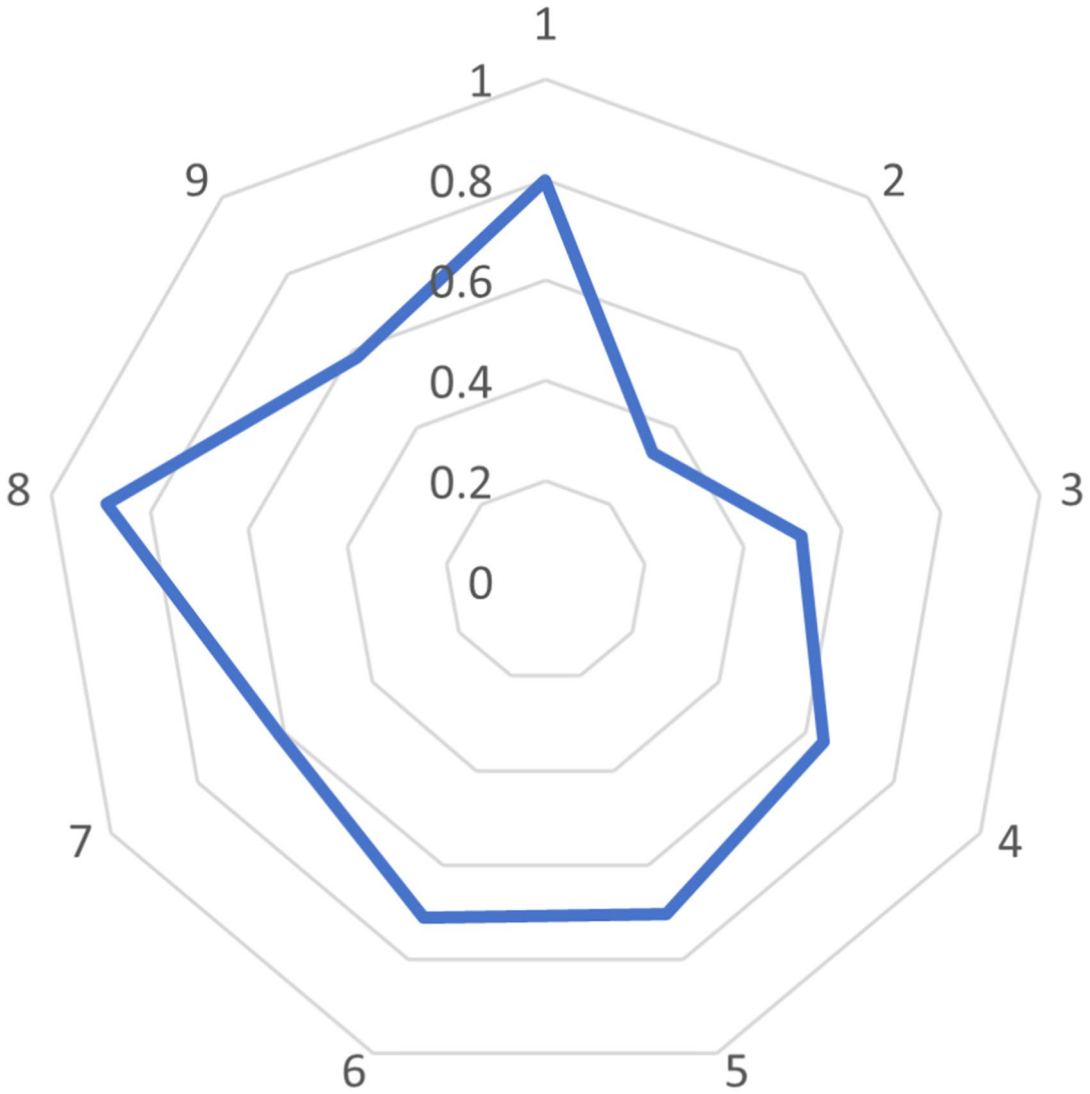
Radar chart of the mean PMC values of the sample policies.

As can be seen from the radar chart of policy means (Figure 10), two indicators, P1 and P8, perform exceptionally well, reflecting that the government attaches great importance to the science, rationality, and effectiveness of policies in formulating policies on digital rural public sports services. In contrast, P2, P3, P4, P7, and P9 have greater concavity and need further improvement.

## 6 Result analysis

Policy P1 Action Plan for Strengthening Efforts to Make up for Weaknesses, Reinforce Key Areas, and Improve the Quality of Public Services in the Social Field to Promote the Formation of a Strong Domestic Market: Policy P1 focuses on addressing deficiencies in rural public services, particularly by leveraging modern technologies such as big data and cloud computing to enhance service quality in the construction and accessibility of public sports facilities. The policy demonstrates strong performance in terms of its nature and functional orientation. However, it scores below average in the areas of incentive mechanisms and policy failure response, which undermines its effectiveness and long-term sustainability. Based on the degree to which each indicator falls below the average, it is recommended to first strengthen the incentive mechanism (Y7) to boost the motivation of grassroots implementation units. Second, the monitoring and response system for policy failure (Y2) should be enhanced to ensure policy continuity. The suggested order of improvement is Y7 → Y2 → Y8.

Policy P2 Basic Public Service Standards for National Fitness (2021 Edition): Policy P2 aims to standardize basic public services for national fitness, with clearly articulated requirements—particularly for rural sports facilities and services—to promote equitable and consistent service delivery. The policy performs exceptionally well in the dimensions of target audiences and key policy priorities. However, it scores below the average in terms of the authority of the issuing body and the diversity of policy tools. Based on the extent to which each indicator lags behind the average, it is recommended to first strengthen the authority and inter-agency coordination capacity of the issuing institution (Y3), and then diversify the range of policy tools (Y9) to support broader implementation of rural sports initiatives. The suggested improvement sequence is: Y3 → Y9 → Y5.

Policy P3 Law of the People’s Republic of China on Promoting Rural Revitalization: Policy P3, as a legal safeguard, incorporates rural public sports facilities into the overall rural revitalization strategy, emphasizing long-term development and sustained investment in rural sports services. While the policy performs well in terms of strategic positioning, it scores below average in the dimensions of incentive mechanisms and policy tools, which limits its practical implementation capacity. Based on the extent to which each indicator falls below the average, it is recommended to first strengthen incentive mechanisms (Y7) to ensure the active engagement of grassroots implementation units. Additionally, enriching the diversity of policy tools (Y9) will help broaden the scope and effectiveness of rural sports services. The recommended order of improvement is: Y7 → Y9 → Y8.

Policy P4 Opinions on Building a Higher-level Public Service System for National Fitness: Policy P4 is committed to building a high-level public service system for national fitness, especially in improving and popularizing rural sports facilities. The policy scores relatively high in the dimensions of the issuing authority and key points. However, it scores lower than the average regarding the coverage of policy audiences and the richness of policy tools. Based on the degree to which each indicator is lower than the average, it is recommended first to clarify the scope of policy audiences (Y4) to ensure that more rural people are covered. Secondly, the policy tools (Y9) should be enriched to improve the implementation effect and diversity of the policy. The improvement order is Y4 → Y9 → Y6.

Policy P5 Guiding Opinions on Promoting the High-quality Development of Farmers’ Sports during the 14th Five-Year Plan Period: Policy P5 focuses on improving the quality of sports services in rural areas during the 14th Five-Year Plan period, improving facilities and service supply, and realizing the equalization of rural sports services. The policy performs prominently in the dimensions of policy focus and functions. However, it scores lower than the average in terms of incentive measures and policy audiences, which affects the promotion and coverage of the policy. Based on the degree to which each indicator is lower than the average, it is recommended first to enhance the incentive measures (Y7), then expand the coverage of policy audiences (Y4) to ensure that more rural residents can enjoy high-quality sports services. The improvement order is Y7 → Y4 → Y8.

Policy P6 Notice of the General Office of the State Council on Printing and Issuing the 14th Five-Year Plan for the Construction of Urban and Rural Community Service Systems: Policy P6 is committed to the construction of urban and rural community service systems to achieve the equalization of urban and rural public sports services. It performs well in the dimensions of policy evaluation and audiences. However, the scores in the prevention of policy failure and the diversity of tools are lower than the average, which limits the continuous effect of the policy. Based on the degree to which each indicator is lower than the average, it is recommended first to strengthen the preventive measures for policy failure (Y2), then enrich the policy tools (Y9) better to meet the diverse sports needs of rural communities. The improvement order is Y2 → Y9 → Y6.

Policy P7 Guiding Opinions on Promoting the Work of Sports Assisting Rural Revitalization: Policy P7 promotes rural revitalization through sports, promotes the development of national fitness in rural areas, and improves the utilization rate and coverage rate of public sports facilities. The policy performs excellently regarding policy nature, key points, and functions. However, the score in the dimension of incentive measures is lower than the average, affecting the policy’s promotion strength. Based on the degree to which each indicator is lower than the average, it is recommended that incentive measures (Y7) be enhanced to encourage the active participation of grassroots implementation subjects. The improvement order is Y7 → Y5 → Y8.

Policy P8 Guiding Opinions on Accelerating the Digital Empowerment of Life Services: Policy P8 focuses on the digital empowerment of life services, including the digital construction of rural sports services. The policy scores are relatively high in terms of functions and tools. However, it scores lower than the average in the dimensions of policy audiences and issuing authorities, which affects the wide promotion of the policy. Based on the degree to which each indicator is lower than the average, it is recommended first to enhance the authority of the issuing authority (Y3) and then expand the scope of policy audiences (Y4) to improve rural residents’ acceptance of digital sports services. The improvement order is Y3 → Y4 → Y9.

Policy P9 Digital Rural Construction Guide 2.0: Policy P9 is an important guiding document for digital rural construction, providing a blueprint for the digital development of public sports services in rural areas. The policy scores are relatively high regarding policy nature and incentive measures. However, it scores lower than the average in the dimensions of policy evaluation and tool diversity, which affects the actual effect of the policy. Based on the degree to which each indicator is lower than the average, it is recommended first to improve the policy evaluation mechanism (Y6) and then enrich the policy tools (Y9) to ensure the policy’s implementation effect and feedback mechanism. The improvement order is Y6 → Y9 → Y8.

## 7 Conclusions and recommendations

First, it is recommended to refine the incentive mechanism to stimulate the enthusiasm and innovative capacity of stakeholders at all levels to actively engage in rural sports services. Policies should adopt rural sports participation rates, facility utilization rates, and service satisfaction levels as core evaluation indicators. Based on these, targeted incentives—such as financial subsidies, preferential resource allocation, and performance-linked rewards—can be introduced. Furthermore, policy outcomes should be incorporated into the performance evaluation systems of local governments and grassroots organizations to establish a multi-tiered, continuous incentive framework. At the same time, policy tools should reflect the unique characteristics of rural sports and adopt modular designs that accommodate the diverse needs of different rural areas. This approach would support more context-sensitive implementation and ensure that service delivery aligns with local conditions and demand.

Second, the formulation and coordination of policies should be further systematized and reinforced with stronger institutional authority. It is recommended to establish an efficient and integrated policy coordination mechanism, positioning rural public sports services as a foundational element in the broader framework of digital rural development. Such a mechanism should ensure the accurate delivery of policy directives from central to local levels, and enhance coordinated efforts in areas such as resource allocation, financial investment, and talent cultivation. To this end, an interdepartmental digital management platform should be developed, integrating resources from the fields of sports, culture, and public health. This platform would facilitate data sharing and information exchange, reduce policy fragmentation, and promote the effective coordination of public services. For example, the platform could issue customized sports promotion programs based on local needs, while simultaneously tracking facility usage and service outcomes to ensure targeted resource investment and efficient policy implementation.

Third, the scientific foundation of policy design and evaluation, as well as the responsiveness of dynamic adjustment mechanisms, must be further strengthened to better align with the evolving needs of rural sports services. It is recommended to establish a systematic and data-driven evaluation framework, using indicators such as facility utilization rates, public participation levels, and the frequency and quality of sporting events. This system should incorporate real-time data tracking and analysis to monitor policy performance and enable timely adjustments as needed. In parallel, a feedback mechanism at the grassroots level is essential. By collecting input directly from rural sports administrators and residents, policymakers can gain insights into local implementation challenges and adapt strategies accordingly. Practical feedback from on-the-ground experiences can help uncover policy blind spots and offer a solid empirical basis for refinement. By improving both feedback loops and policy adjustment mechanisms, rural sports policies can become more flexible, targeted, and effective—ensuring steady improvements in service delivery while injecting sustained momentum into broader rural revitalization efforts.

This study is closely aligned with the goals of sustainable development in several important respects. First, the long-term sustainability of policy implementation is a core concern. Although current policies have yielded some progress, they still fall short in terms of effectiveness, coverage, and durability. Without strong and lasting implementation mechanisms, the advancement of rural public sports services remains limited. Second, the integration of digital technologies holds significant promise for optimizing resource allocation and promoting the sustained development of rural sports services. By improving access and facilitating resource sharing, digital platforms enhance both service efficiency and equity. Their application not only expands the reach of public sports services but also strengthens their long-term viability, injecting new energy into rural development and serving as a key driver of sustainability. Finally, building a collaborative, cross-sectoral policy framework is essential to ensure the effectiveness and continuity of these efforts. Such a mechanism promotes balanced access to sports services across urban and rural areas, fosters social cohesion, and contributes to the overall well-being and sustainable growth of rural communities.

## 8 Future directions and discussion

While this study attempts to evaluate digital rural public sports service policies using the PMC index model—achieving a degree of structural consistency analysis and visual interpretation—it also presents certain methodological limitations that point to valuable directions for future exploration.

To begin with, the current use of binary (0–1) coding for variable assignment enhances the model’s comparability and operational clarity. However, it inevitably simplifies the semantic nuance and contextual depth of the original policy texts. Future research could address this by adopting multi-level scoring scales, fuzzy set theory, or expert-weighted mechanisms to improve the granularity and interpretive strength of policy coding. In addition, differentiated weighting schemes—such as the Analytic Hierarchy Process (AHP) or entropy-based methods—could be introduced to assign variable importance more realistically, thereby improving the model’s alignment with actual policy significance.

Second, the PMC model, as a structural analytical framework, does not directly reflect real-world policy outcomes or implementation data. This methodological boundary could be bridged by integrating complementary tools such as the Logical Framework Approach (LFA), Data Envelopment Analysis (DEA), or field-based survey data to construct a closed-loop evaluation system encompassing policy design, execution, and feedback.

Third, although this study employed a Delphi-based expert consultation process to ensure the relevance, completeness, and timeliness of selected policy samples, future research might benefit from broadening its scope. Cross-regional comparative analyses or multi-tiered policy studies could enrich the empirical base and provide more diverse insights into implementation variability.

Lastly, in the domain of digital rural public sports, the success of policy implementation is often constrained by local realities such as infrastructure availability, personnel training, and funding capacity. While textual analysis provides a valuable window into policy intentions, it may not fully capture on-the-ground effectiveness. Therefore, future studies are encouraged to combine policy document analysis with field investigations, monitoring data, and simulation modeling, enabling a more integrated assessment that moves from “well-formulated” to “well-implemented” policy.

## Data Availability

The original contributions presented in the study are included in the article/supplementary material, further inquiries can be directed to the corresponding author.
